# A Gene Module-Based eQTL Analysis Prioritizing Disease Genes and Pathways in Kidney Cancer

**DOI:** 10.1016/j.csbj.2017.09.003

**Published:** 2017-10-10

**Authors:** Mary Qu Yang, Dan Li, William Yang, Yifan Zhang, Jun Liu, Weida Tong

**Affiliations:** a Joint Bioinformatics Graduate Program, Department of Information Science, George W. Donaghey College of Engineering and Information Technology, University of Arkansas at Little Rock, USA; b University of Arkansas for Medical Sciences, 2801 S. University Ave, Little Rock, AR 72204, USA; c School of Computer Science, Carnegie Mellon University, 5000 Forbes Ave, Pittsburgh, PA 15213, USA; d Department of Statistics, Harvard University, Cambridge, MA 02138, USA; e Divisions of Bioinformatics and Biostatistics, National Center for Toxicological Research, US Food and Drug Administration, 3900 NCTR Road, Jefferson, AR 72079, USA

**Keywords:** RCC, Renal cell cancer, ccRCC, Clear cell renal cell carcinoma, eQTL, Expression quantitative trait loci, SVM, Support vector machine, TCGA, The Cancer Genome Atlas, KEGG, Kyoto Encyclopedia of Genes and Genomes, DEG, Differentially expressed gene, DGM, Differential gene module, AUC, Area Under Curve, ROC, Receiver Operating Characteristic, ccRCC, Causative mutation, Pathways, Protein-protein interaction, Gene module, eQTL

## Abstract

Clear cell renal cell carcinoma (ccRCC) is the most common and most aggressive form of renal cell cancer (RCC). The incidence of RCC has increased steadily in recent years. The pathogenesis of renal cell cancer remains poorly understood. Many of the tumor suppressor genes, oncogenes, and dysregulated pathways in ccRCC need to be revealed for improvement of the overall clinical outlook of the disease. Here, we developed a systems biology approach to prioritize the somatic mutated genes that lead to dysregulation of pathways in ccRCC. The method integrated multi-layer information to infer causative mutations and disease genes. First, we identified differential gene modules in ccRCC by coupling transcriptome and protein-protein interactions. Each of these modules consisted of interacting genes that were involved in similar biological processes and their combined expression alterations were significantly associated with disease type. Then, subsequent gene module-based eQTL analysis revealed somatic mutated genes that had driven the expression alterations of differential gene modules. Our study yielded a list of candidate disease genes, including several known ccRCC causative genes such as *BAP1* and *PBRM1*, as well as novel genes such as *NOD2, RRM1, CSRNP1, SLC4A2, TTLL1* and *CNTN1.* The differential gene modules and their driver genes revealed by our study provided a new perspective for understanding the molecular mechanisms underlying the disease. Moreover, we validated the results in independent ccRCC patient datasets. Our study provided a new method for prioritizing disease genes and pathways.

## 1 Introduction

Kidney cancer is the sixth most common form of cancer for men and the tenth most common form of cancer for women. In 2016, over 63,000 newly diagnosed cases and 14,400 kidney cancer deaths were reported in the United States [1]. The vast majority of kidney cancers are renal cell carcinomas (RCC), among which nearly 75% are clear cell renal cell carcinomas (ccRCC) [2]. Despite recent advances, metastatic RCC remains largely an incurable disease [3], [4]. Patients with this disease often have no apparent symptoms or laboratory abnormalities in the early stages. The incidence of ccRCC has been rising steadily in recent years due to the prevalence of adverse lifestyle changes and exposure to toxins such as smoke [5].

ccRCC is characterized by the presence of *VHL* gene mutation in most cases [6]. However, the loss of *VHL* alone is not sufficient for tumor initiation and survival, and a fraction of ccRCCs contain wild-type *VHL* genes, suggesting additional genetic alterations are required in the course of tumor development. Recent large-scale sequencing studies of ccRCC, including TCGA (The Cancer Genome Atlas) project have discovered several new and prevalent genomic mutations such as *PBRM1* and *BAP1* [7], [8], [9]. Despite these findings, the mortality rate of ccRCC has not significantly decreased, indicating that the genetic basis of the disease occurrence and development remains to be elucidated. Additionally, previous studies have shown that ccRCC is a highly heterogeneous disease [10], [11], creating the need to identify new disease genes and pathways.

The expression quantitative trait loci (eQTL) analysis has been used to identify single-nucleotide polymorphisms (SNPs) that are significantly associated with gene expressions [12], [13], [14]. Most eQTL analysis performed testing on transcript-SNP pairs to identify genetic mutations that significantly affected individual gene expression. Here, we presented a gene module-based eQTL method to identify the somatic mutations that are associated with gene clusters, which potentially function in the same pathway. We first identified differentially expressed gene modules (DGMs). The DGMs are comprised of a set of interacting genes based on protein-protein interactions and expression profile. The Gene Ontology analysis suggested that majority DGMs contained genes involved in the same biological processes. Additionally, the genes inside the same DGM tended to be co-expressed. Hence, these gene modules most likely contained genes function together in the disease-affected pathway. Disease genes are not always differentially expressed. The integration of gene expressions and protein interactions empower the discovery of disease genes, as disease genes without significant expression alterations could be revealed by DGMs through interacting with the differentially expressed genes in the gene modules. The subsequent eQTL analysis further established the linkages of somatic mutations with the DGMs. Collectively, the DGMs and their associated genetic mutations lead to the identification of novel disease genes and pathways. Moreover, we examined the DGMs on four independent ccRCC patient cohorts. The results showed DGMs accurately classified the tissue types blindly.

## 2 Results

**Fig. 1:**
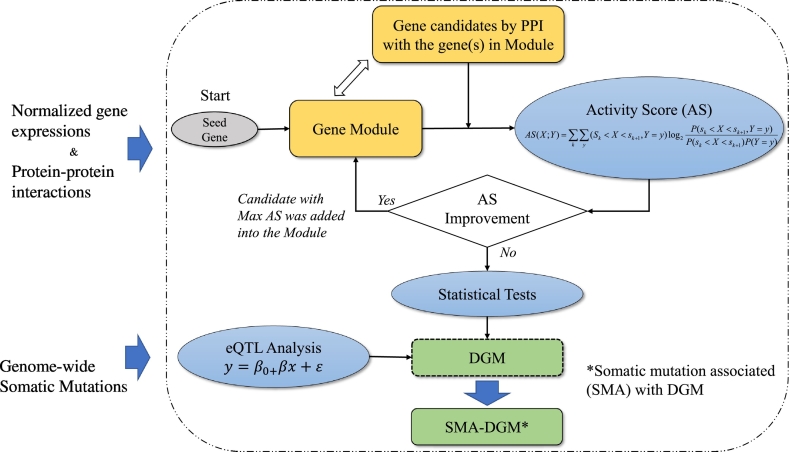
The procedure of our study. After the differentially expressed gene modules were identified by coupling PPI with gene expression, somatic mutations were linked with the DGMs using eQTL analysis. Here, SMA-DGM refers to somatic mutations associated the DGMs.

An interacting pathway regulates the expression of a group of genes that often perform certain functions together. When a pathway is perturbed by genetic mutations, then expression levels of interacting genes associated with the pathway can be altered accordingly and can further contribute to malignant transformation. By integrating gene expression and protein-protein interactions here, we developed a new method of identifying gene clusters in the pathways impacted by the disease. Then, we performed an eQTL analysis to infer potential driver mutations and disease affected pathways. The procedure of our study was illustrated in Fig. 1.

### 2.1 Differentially Expressed Gene Modules Identification

The RNA-Seq expression profile of 19,768 protein-coding genes was obtained from TCGA 539 ccRCC and 72 paired normal tissue samples. After filtering out the genes with very low expression levels (Methods), a total of 16,343 genes remained for the subsequent analysis. Then, we coupled gene expression and protein-protein using a network approach to systematically reveal gene modules that were differentially expressed in ccRCC. At first, each individual gene was employed as the seed of a module, and new genes were added to the module in an iterative manner. At each step, all genes that interacted with any gene member of the module were assessed using an activity score. A higher activity score suggested the expression level of the corresponding module was more likely associated with the tissue phenotype (Methods, Fig. 1). Hence, the gene that maximized the activity score was selected and added to the module. After a gene module was built, we applied three statistical tests to evaluate the significance of the module compared to background. The three tests included permuting tissue phenotype, randomizing genes in the module, and randomizing genes in the module with the same seed protein, respectively (Methods). Finally, we identified 1066 significant gene modules with activity scores equal or larger than 0.34 (*P*-value < 0.001 in all three statistical tests, Fig. 1). We referred to these gene modules as differential gene modules (DGMs).

### 2.2 Performance Evaluation Classification Based on Differential Gene Modules

The DGMs represented gene clusters that were significantly associated with tissue phenotypes. Thus, we hypothesized that the expression levels of DGMs can be utilized as features to distinguish ccRCC tissue from normal tissue samples. We examined the hypothesis using the TCGA-ccRCC dataset and three independent ccRCC patient datasets obtained from GEO (Methods, Table 1) [4], [9]. The TCGA dataset contained an imbalance between ccRCC and normal samples (539 ccRCC versus 72 normal samples), whereas the other three data sets contained more balanced samples (Table 1).

**Table 1:** The performance of DGM and DEG based hierarchy clustering and SVM classifiers on the TCGA ccRCC patient group and three independent ccRCC datasets.

ccRCC patient cohorts	Normal	Tumor	Misclustered tissue samples		AUC of the classifiers	
			DGM-based	DEG-based	DGM-based	DEG-based
TCGA-ccRCC	72	539	2	4	**0.942**	**0.767**
GSE36895	23	29	0	0	0.923	1.0
GSE46699	63	67	9	15	0.953	0.949
GSE40435	101	101	0	0	0.956	0.997

The DGM based classifier significantly outperformed the DGE based classifier by 22.8%((0.942 -0.767) / 0.767, Table 1 bold number) on the TCGA-ccRCC which is an imbalanced data set (72 normal vs 539 tumor samples).

The differentially expressed genes (DEG) based evaluation was performed for comparison as well. The TCGA expression profile was generated using RNA-Seq data, whereas the expression profiles of the other three independent ccRCC patient cohorts were produced using Microarray data (Methods). We used edgeR for the TCGA RNA-Seq dataset, and *t*-test followed by multiple-test correction for microarray datasets to perform differential expression analyses (Methods, Supp. Fig. 1).

**Fig. 2:**
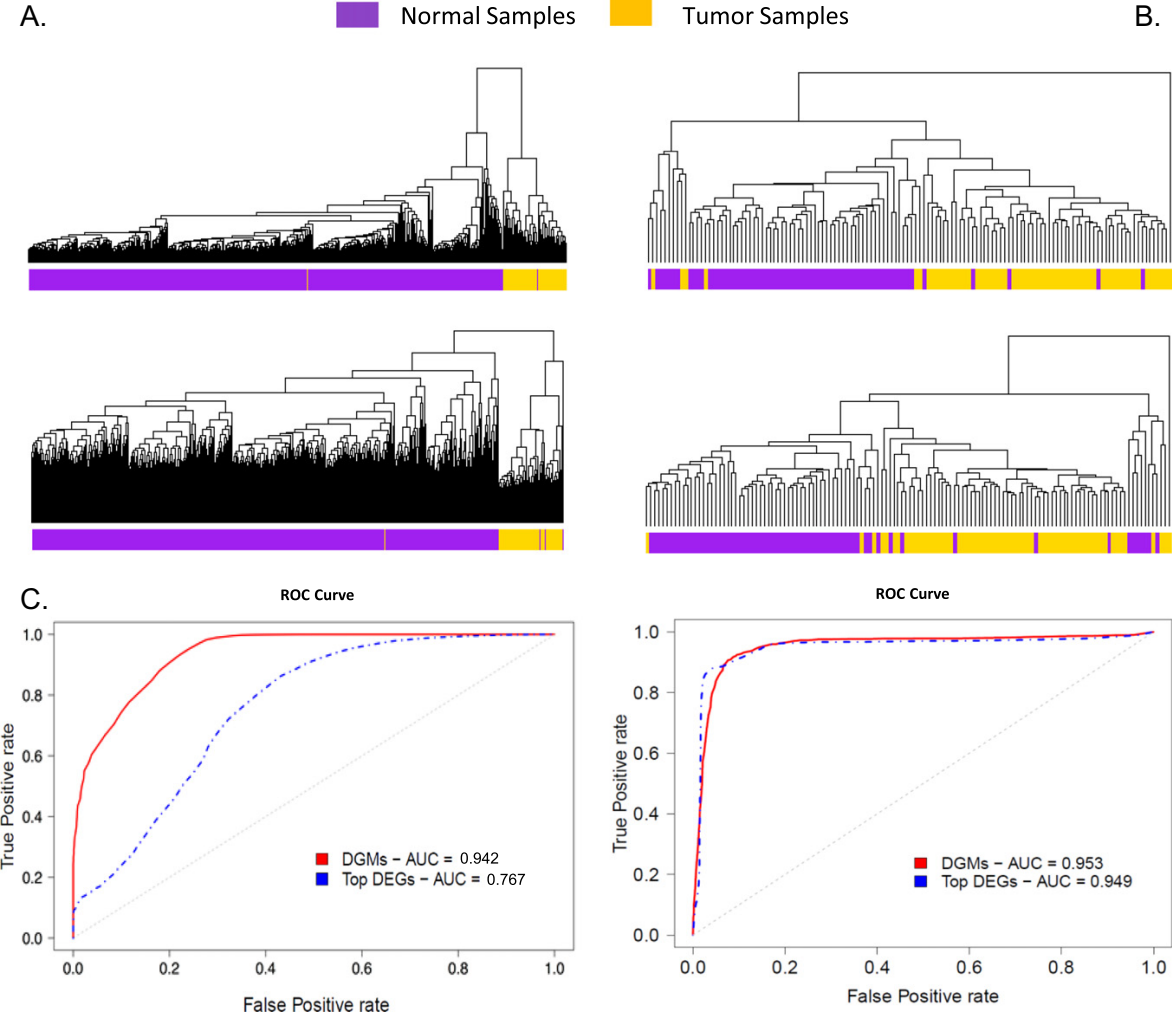
The performance comparisons of clustering and classification based on DGMs and DEGs. (A) Hierarchical clustering of TCGA 539 ccRCC and 72 normal tissues based on the expression of DGMs (top) and DEGs (bottom). (B) Hierarchical clustering of an independent ccRCC (GSE46699) tumor and normal tissues based on DGMs (top) and DEGs (bottom). (C) The ROC curves of the classifiers using the expression of DGM and DEG for the TCGA dataset locate at left panel, for GSE46699 locate at right panel.

We conducted hierarchical clustering analysis*,* using DGMs and DEGs, respectively, on the four ccRCC datasets including the TCGA dataset, GSE36895, GSE40435 and GSE46699. For GSE36895 and GSE40435, both using DGMs and DEGs yielded distinctive tumor and normal tissue clusters with perfect homogeneity (Table 1). However, for the TCGA data and GSE46699, the clusters yielded by DGMs tended to be more homogeneous as compared to the clusters generated by the DEGs. Four tumor tissue samples were misclustered using DEGs (Fig. 2A top panel, Table 1), whereas the number of misclustered tumor tissues was reduced to two using DGMs for the TCGA data (Fig. 2A bottom panel, Table 1). For the GSE46699, using DGM resulted in 9 misclustered tumor samples (Fig. 2B top panel, Table 1), whereas using DGE yielded 15 misclustered ccRCC samples (Fig. 2B bottom, Table 1).

Moreover, we built SVM-based (Support Vector Machine) classifiers to predict tissue type using the expression levels of DGMs and DEGs genes as input features, respectively. The area under curve (AUC) of the receiver operating characteristic (ROC) curve generated by three-fold validation was measured for classification performance assessment. The AUC of the classifier using DGM as features is 0.942, which is significantly higher than 0.767 for the classifier using DEG as features, in predicting TCGA dataset tissue types (Fig. 2C left panel). However, additional classifications on three independent ccRCC datasets, which included fewer but more balanced tissue samples than the TCGA dataset, showed that the DEG-based classifiers performed slightly better or very similar compared to the performance of DGM based classifiers (Table 1, Fig. 2C right panel). Nevertheless, the DGM yielded good performance more robustly in both classification and clustering and suggests that genes in the differential modules were significantly associated with ccRCC; they are coordinately expressed; and, they likely function together in the disease pathways.

### 2.3 Functional Assessment of the Gene Modules

We conducted Gene Ontology (GO) analysis on individual DGMs. The GO terms enrichment of were assessed by hypergeometric test (*P* < 0.01). Given that the median size of the gene modules is 6, we found that 90.4%, 65.4% and 39.9% (963/1065, 696/1065, and 425/1065) of these modules contained at least two, three, and four genes that participated in the same significantly enriched biological process, respectively. In contrast, none of the random modules that had the same topology and size as the DEMs contained more than one gene in the same biological process.

Additionally, our expression analysis showed that the majority of the genes in the DGMs appeared to be co-expressed (74.5%, 793/1065). Thus, the significant modules more likely consisted of genes functioning together in the disease-related pathways.

We found a total of 22 enriched biological process terms that were significantly associated with at least 18.9% (201/1065) of the DGMs (Supp. Table 1), including several known cancer-related biological processes. For instance, 209 gene modules were prevalent in the neurotrophin Tropomyosin Receptor Kinase (TRK) receptor signaling pathway, a pathway involving malignant gliomas [15].

Moreover, we identified 26 Kyoto Encyclopedia of Genes and Genomes (KEGG) pathways that were significantly enriched in at least 8.5% (90/1065) of the gene modules (Supp. Table 2). Thyroid cancer pathway, a top-affected pathway in our list, was significantly associated with 27.9% (297/1065) of gene modules (*P* < 0.05, hypergeometric test). It has been reported that ccRCC is most frequent of origin of thyroid metastases and represents 12 to 34% of all secondary thyroid tumors [16], [17], [18]. 18.4% (196/1065) modules included genes that are significantly prevalent in fatty acid degradation pathways. Cellular proliferation requires fatty acids for synthesis of membranes and signaling molecules. Dysregulation of cellular proliferation is associated with the occurrence of cancer.

### 2.4 Gene Module-based eQTL Analysis

**Fig. 3:**
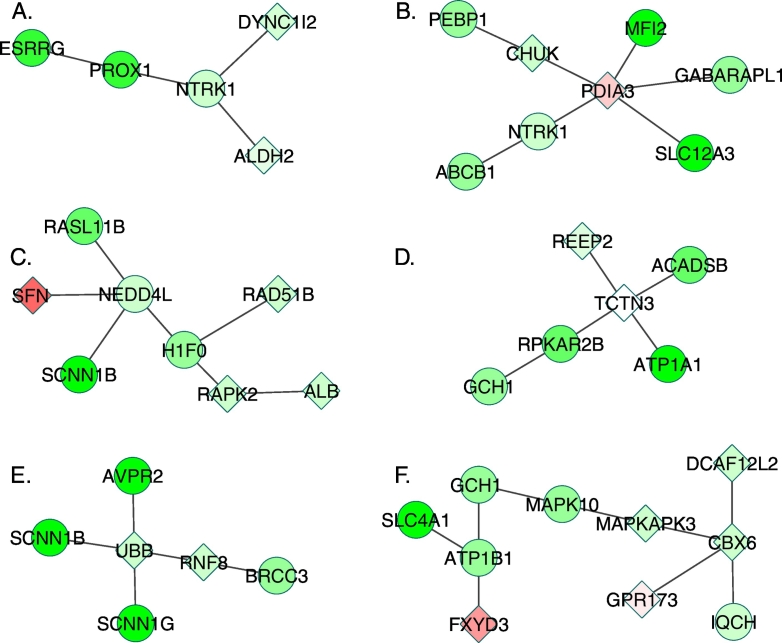
The examples of differentially expressed gene modules. The genes colored in red were up-regulated, whereas genes colored in green were down-regulated in ccRCC. The intensity of the color is proportioned to log2 fold-change of the gene expression. Circle nodes refer to the expression levels of genes that were significantly changed, whereas diamond nodes refer to the genes without significantly altered expression levels. (For interpretation of the references to color in this figure legend, the reader is referred to the web version of this article.)

We performed eQTL analysis on differential gene modules. The mutation of *VHL*, a known ccRCC causative gene, was found to be significantly associated with multiple DGMs (FDR < 0.03, Fig. 3A and B). These modules were enriched of genes in MAPK signaling pathway, apoptosis, pathways in cancer (*P* < 0.03, hypergeometric test).

To further prioritize the most significant somatic mutations that were associated with the differential gene modules, we employed FDR < 0.0001 as the cutoff in the eQTL analysis. The number of somatic mutations that were significantly associated with each differential module was assessed. Overall, we found 780 of 1065 modules were significantly associated with at least one somatic mutation. The median number of associated somatic mutations with DGMs is 8 (Supp. Fig. 2). 188 modules were significantly associated with five or less somatic mutations (Table 2). Some mutated genes influenced many DGMs.

**Table 2:** A total of 188 DGMs were significantly associated with five or less mutated genes (FDR < 0.0001).

Num. of mutated gene(s) associated with each DGM	Num. of associated DGMs	Mutated genes associated with the DGMs⁎
1	116	***BAP1*(40**), *NOD2*(18), ***RRM1*(13),***CSRNP1*(8), ***PBRM1*(8),***CNTN1*(5), *PGM5*(3), *RBM27*(2), *FAM19A1*(2), *KCNT2*(2), *PHKB*(2), *ZNF624*(2), *MARCH1*(1), *CACNA1E*(1), *IPO4*(1), *PLXNA2*(1), *SLC12A9*(1), *SLC15A4*(1), *SSRP1*(1), *TACC1*(1), *UBN2*(1), *ZNF844*(1)
2	47	***BAP1*(23), *PBRM1*(13), *RRM1*(11),***NOD2*(9), *SLC4A2*(9), *TTLL1*(9), *CSRNP1*(2), *PGM5*(2)*, PHKB*(2)*, March1*(1)*, ATG3*(1), *ATG4C*(1), *CD180*(1), *CNTN1*(1), *EPHA1*(1), *EVPL*(1)*, LAMB4*(1), *PARD6A*(1), *RBM26*(1), *SETD2*(1), *SPTBN1*(1), *TMEM17*(1), *ZNF711*(1)
3	16	***BAP1*(12), *PBRM1*(8),***CNTN1*(4), *CSRNP1*(4), *PHKB*(3), *SLC4A2*(3), *TTLL1*(3), *CHD8*(2), *FAM19A1*(2), *NOD2*(2), *CACNA1E*(1), *FRS2*(1), ***RRM1*(1),***SPTBN1*(1), *UBA7*(1)
4	4	***PBRM1*(5), *BAP1*(4),***CAST*(1), *CD200R1*(1), *COL14A1*(1), *GRM3*(1), *NCOA5*(1), *PCOLCE2*(1), *ZNF572*(1)
5	5	*MEIS3*(3), *MOB3B*(3), *R3HCC1*(3), *SHISA5*(3), *WEE1*(3), *CAST*(2), *CD200R1*(2), *COL14A1*(2), *PCOLCE2*(2), ***BAP1*(1), *RRM1*(1)**

⁎ The number in the parentheses after a gene symbol represents the number of the DGMs that were linked with this mutated gene.

**Fig. 4:**
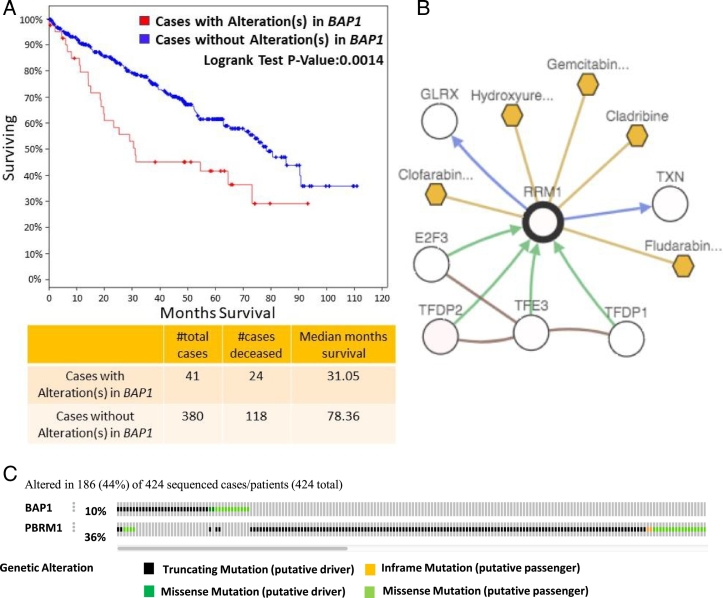
The analysis of the genes harbored significant somatic mutations and were associated with the DGMs. (A) The ccRCC patients with *BAP1* somatic mutations had poor five-year survival rate. (B) Five FDA-approved cancer drugs (colored in gold) target at RRM1. (C) The mutation of BAP1 and PBRM1 tend to mutual exclusively at *P* < 0.03.

*BAP1* and *PBPM1* mutations significantly impacted 42.5% (80 of 188) and 18.1% (34/188) of the DGMs (Table 2). *BAP1* loss has been reported to define a new class of ccRCC and acts as a tumor suppressor [4]. In addition, ccRCC patients with *BAP1* somatic mutations had poor 5-year survival rates (*P* < 0.014, Fig. 4A). *PBRM1* encodes a protein that changes chromatin structure and influences p53 transcriptional activity. The previous study suggested that PBRM1 protein is regulated by p53-induced protein degradation in renal cell carcinomas [19]. Interestingly, the mutations of somatic mutations of *BAP1* and *PBRM1* tend to be mutually exclusive (*P* < 0.03, Fisher Exact Test, Fig. 4C). *RRM1*, a target of five Food and Drug Administration (FDA) approved cancer drugs, were significantly associated with 13.8% (26/188) of differential modules. *RRM1* is involved in carcinogenesis, tumor progression, and induces metastasis suppression though PTEN-regulated pathways [20], [21].

The other somatic mutated genes that significantly affected at least 5.3% of differential gene modules included *NOD2, RRM1, CSRNP1, SLC4A2, TTLL1* and *CNTN1.* The genetic alterations of these eight genes were significantly associated with poor survival rates of ccRCC patients (*P* < 0.054, Supp. Fig. 3). However, the mutations only presented in 2.6% (11/421) TCGA ccRCC patients. The association of the genetic mutations and survival rage need to be interpreted with caution. Presently, the functional roles of eight genes in ccRCC have not yet been studied. Collectively, our results indicated that the gene module-based eQTL analysis yielded a list of putative disease genes, including known ccRCC genes such as *VHL*, *BAP1*, and *PBRM1,* as well as novel disease genes.

## 3 Methods

### 3.1 The Whole-exome and Transcriptome Data of ccRCC Patients

The whole-exome sequencing and RNA sequencing data were obtained from the TCGA data portal. The tumor and paired normal tissue samples were collected from newly diagnosed ccRCC patients having no prior treatment for this disease, including chemotherapy or radiotherapy. The sequencing data were generated using Illumina Hiseq 2000 pair-end sequencing. The ccRCC patients consisted of 65% male, 35% females, and represented 93% Caucasian, 3.4% African American/Black, and 1.6% Asian. The median age of patients at diagnosis is 60.9 years [9]. The whole-exome sequencing reads from 417 of paired tumor and normal tissue samples were aligned to human reference genome using Blat-like Fast Accurate Search Tool (BFAST) [22]. Then, genome-wide somatic mutations were detected using the MuTect algorithm [23]. The RNA sequencing data was generated from 539 tumor and 72 matched normal tissue samples. After poor quality reads were removed using the srf2fastq tool (Staden package), the RNA sequencing reads were aligned to reference transcript database using BWA algorithm [24]. The genes that have the mean Fragments Per Kilobase of transcript per Million mapped reads (FPKM) values < 0.1 in tumor samples as well as normal samples were removed from the expression profile. The differential expressed genes were detected using edgeR package in R [25]. The output of edgeR includes fold change and false discovery rates for individual genes. The gene that satisfied two criteria simultaneously, FC > 2 and FDR < 0.01, were considered as differentially expressed genes.

We also attained three independent ccRCC microarray datasets from the Gene Expression Omnibus database (GEO) for validation. The first dataset (GSE36895) contained 23 normal and 29 tumor tissue samples of a group of ccRCC patients [4]. The second dataset (GSE46699) contained 65 normal and 65 ccRCC paired samples of individual patients [26]. The third dataset (GSE40435) contained 101 normal and 101 paired ccRCC tissue samples [27]. The microarray data was generated using Affymetrix Human Genome U133 Plus 2.0 arrays. We applied *t*-test followed by Benjamini-Hochberg multiple test correction to identify the differentially expressed genes in ccRCC.

### 3.2 Protein-protein Interactions

Protein-protein interaction (PPI) data were obtained by combining five public PPI databases: intAct, MINT, BioGrid, DIP, and Reactome. The intAct database is quite comprehensive, containing information from 12 databases such as MINT, UniPort, mpidb, etc. Only human PPIs were selected for our study. For instance, we used “homo_sapiens.mitab.interactions” to attain human PPI in humans in the Reactome database. After filtering the redundancy of the union set of all PPIs, we attained a total of unique 440,747 PPIs. Then, we performed two expression correlation assessments using the expression profile of TCGA ccRCC tumor and normal tissue samples. The first correlation assessment is simply the correlation of the expression levels of the two genes across all tissue samples, while the second is the difference in the expression level correlations of the two genes between tumor and normal tissues. The PPI pairs that rank simultaneously below 5% in both correlation assessments were removed. A total of 319,291 PPIs were retained for differential gene module construction.

### 3.3 Gene Modules Construction

The expression levels of individual genes were normalized across samples by *Z*-score transformation (μ = 0). A gene module started with a seed gene. Then, more genes were added interactively into the module based on PPIs and mutual information assessment. According known PPIs at each step, all genes interacting with any gene members in the current modules were evaluated by mutation information. Mutation information measures the degree to which two random variables are independent. When a random variable *X* is independent of another random variable *Y*, the resulting mutation information is 0. Here, we tested for whether the expression levels of gene modules (X) are associated with tissue types (Y) (ccRCC versus normal). The candidate gene that maximized the mutual information was selected. Here, we referred to the value of mutual information as the activity score. As X is a discrete variable, we discretized normalized expression level (Z) by dividing the range of *Z* into equally spaced bins defined by split points *s_k_*, resulting in the following expression for the activity score calculation:

$$\mathrm{AS}\left(X;Y\right)=\sum_{k}\sum_{y}P\left(s_{k}<X<s_{k+1},Y=y\right)\mathrm{lo}g_{2}\frac{P\left(s_{k}<X<s_{k+1},Y=y\right)}{P\left(s_{k}<X<s_{k+1}\right)P\left(Y=y\right)}$$

At each iterative step, we calculated the improvement of the activity score. The searching procedure was terminated if there is no further improvement by adding new genes into the gene module. Then, we performed three statistical tests to assess the significance of all gene modules. We permuted the tissue phenotype 1000 times to obtain the null distribution in order to test the hypothesis that the gene module is significantly associated with tissue phenotypes. Then, we constructed the other two null distributions by randomly selecting the same number of genes as the gene module retaining seed genes, both with and without seed genes, 1000 times to test the hypothesis that the gene modules are significantly different from the background.

### 3.4 A SVM-based Classifier

A SVM R package “e1071” based on widely used “libsvm” was applied to build the classifier. We adopted “sigmoid” as the kernel function, and default values for all other parameters. The normalized average expression levels of genes in DGMs were used as features for the differential gene module-based classifier.

### 3.5 eQTL Analysis

We used Matrix eQTL to assess the association of somatic mutations and differential gene modules [28]. The linear regression model was adopted in the eQTL analysis. If any gene member in the module had significant associations with the point somatic mutations (FDR < 0.0001), we considered the mutations to be associated with the gene module.

## 4 Discussion

The molecular pathogenesis of many cancer types, including ccRCC, is poorly understood, and can be partially attributed to a limited understanding about comprehensive causative genes and pathways that govern disease initiation and development. The method we developed in this study included two stages: constructing differentially expressed-gene modules and identifying causative mutated genes associated with gene modules. The results yielded by both steps can lead to an expansion of current ccRCC genes and pathways sets.

The differential gene modules were built by coupling known PPIs and expression profiles of ccRCC patients. The number of PPIs has been increasing exponentially in recent years. On the contrary, databases of pathways remain incomplete and largely generic. At present, the majority of the pathways represent summaries of the most conserved components of such pathways and not necessarily what really occurs in each individual case. In addition, pathways can change between tissues, cell types, individuals, and species [29]. Our method offered a way to dynamically discover the ways in which gene clusters function together in the disease state. Gene ontology and pathways enrichment analysis suggested differential gene modules presented a set of genes that function together in the same biological process related to the diseases. Given that the median size of gene modules is six, 90.4% and 45% of gene modules contained at least two genes were significantly associated with biological process and KEGG pathways, respectively. In contrast, none of the random gene modules having the same topology and size as the differential gene modules had two genes associated with the same biological process or pathways. Thus, our results have lead to the discovery pathways involved in ccRCC.

We used normalized expression levels of gene modules as input features to build a SVM-based classifier for predicating tissue types. The classifier achieved over 0.97 AUC in classifying over 600 TCGA ccRCC tissue samples, compared to 0.77 for a SVM classifier using the expression levels of individual genes as input features. The differential gene modules-based classifier achieved over 0.92 AUC for prediction three independent ccRCC patient cohorts (GSE36895, GSE46699, and GSE40435). Thus, the DGMs could be used as molecular signatures to infer tissue phenotypes.

The eQTL has often been applied on transcript-SNP pairs. Here, we implemented the eQTL mapping to the differential gene modules. As the gene modules represent gene clusters in the same pathway, the significant somatic mutations can be linked directly to the disease-affected pathways and suggested potential association between mutations and the pathways. The known ccRCC genes*, BAP1* and *PBRM1*, were revealed by our study. The mutations of *BAP1* and *PRMB1* were the most frequently associated with DGMs (Table 2). The *BAP1* encodes a protein called ubiquitin carboxyl-terminal hydrolase BRCA1-associated protein 1 (BAP1). The BAP1 is associated with multi-protein complex, which regulated several crucial cellular pathways including cell cycle, cell death, the DNA damage response and gluconeogenesis [30]. BAP1 is inactive in 15% of ccRCCs and the loss of BAP1 has defined a new class of ccRCC [31]. The germline mutation of *BAP1* has been associated with high risk of neoplasms [32]. *PRBM1* (Polybromo 1), a SWI/SNF chromatin remodeling complex gene, is frequently mutated in ccRCC [33].

A set of new genes *NOD2, RRM1, CSRNP1, SLC4A2, TTLL1* and *CNTN1,* as well as their associated gene modules, were identified. The mutation of *NOD2* can lead to impaired activation of NFKB in vitro [34] and has associated with colorectal, ovarian and breast cancer [35], [36], [37]. *RRM1* is reported as metastasis suppressor gene by inducing expression of PTEN [38]. Currently, five FDA approved cancer drug target at *RRM1* (Fig. 3). *CSRNP1* involves in apoptotic process and may play a role in apoptosis [39]. *SLC4A2* encodes anion exchanger 2 (AE2**)** and AE2 has been associated with multiple cancer types [40]. CNTN1**,** a protein encoded by *CNTN1*, promoted lung cancer invasive and metastasis [41]. Despite that these genes have been linked to the tumorgenesis of various cancer, their roles in ccRCC have not been extensively studied yet. The genes and their associated DGMs can offer guidance to perform experiments to further validate their functional roles in ccRCC. Thus, our two-stage method provides a new way for identifying new disease genes and their affect pathways.

To date, PPI databases may still contain false positives, e.g., bias in the PPI experiments (some proteins have been studied more than others). Our co-expression assessments may help to reduce the negative effect. Additionally, our results suggested that eQTL analysis could prioritize disease candidate genes, however, true associations may be overlooked and further experimental validation may be needed. The eQTL analysis was based on transcription level, which are quantitative traits relying on accurate and precise measurement of gene expression. Additionally, the sample size may limit the sensitivity for identifying true associations. Similar to the GWAS study, increasing sample size will lead to more association discovery. On the other hand, eQTL analysis may introduce false positives. The significant expression difference between normal and tumor could also attribute to the other somatic alterations such as copy number variations and methylation events. Nevertheless, our study yielded novel candidate genes for further experimental validation, which could potentially advance our understanding of ccRCC.

## 5 Conclusions

Our method integrated whole-exome sequencing data, transcriptome, and PPIs to identify disease genes. These genes harbored somatic mutations that significantly impacted the expression alteration of differential gene modules. The differential gene modules were shown to function in the biological process and their expression levels can be used as molecular signatures to predict unknown tissue types. Our results confirmed several known ccRCC causative as well as novel genes involved in diseases.

## Competing Interests

The authors declare that they have no competing interests.

## Appendix A Supplementary data

Supplementary Figures Image 1

Supplementary Tables Image 2
