# Supplementary material for: A Gene Module-Based eQTL Analysis Prioritizing Disease Genes and Pathways in Kidney Cancer
Source: Comput Struct Biotechnol J. 2017 Oct 10;15:463–70. doi: 10.1016/j.csbj.2017.09.003 (PMC5683705; doi:10.1016/j.csbj.2017.09.003)
Supplement: Supplementary file 1 — Supplementary Figures [file mmc1.pdf]

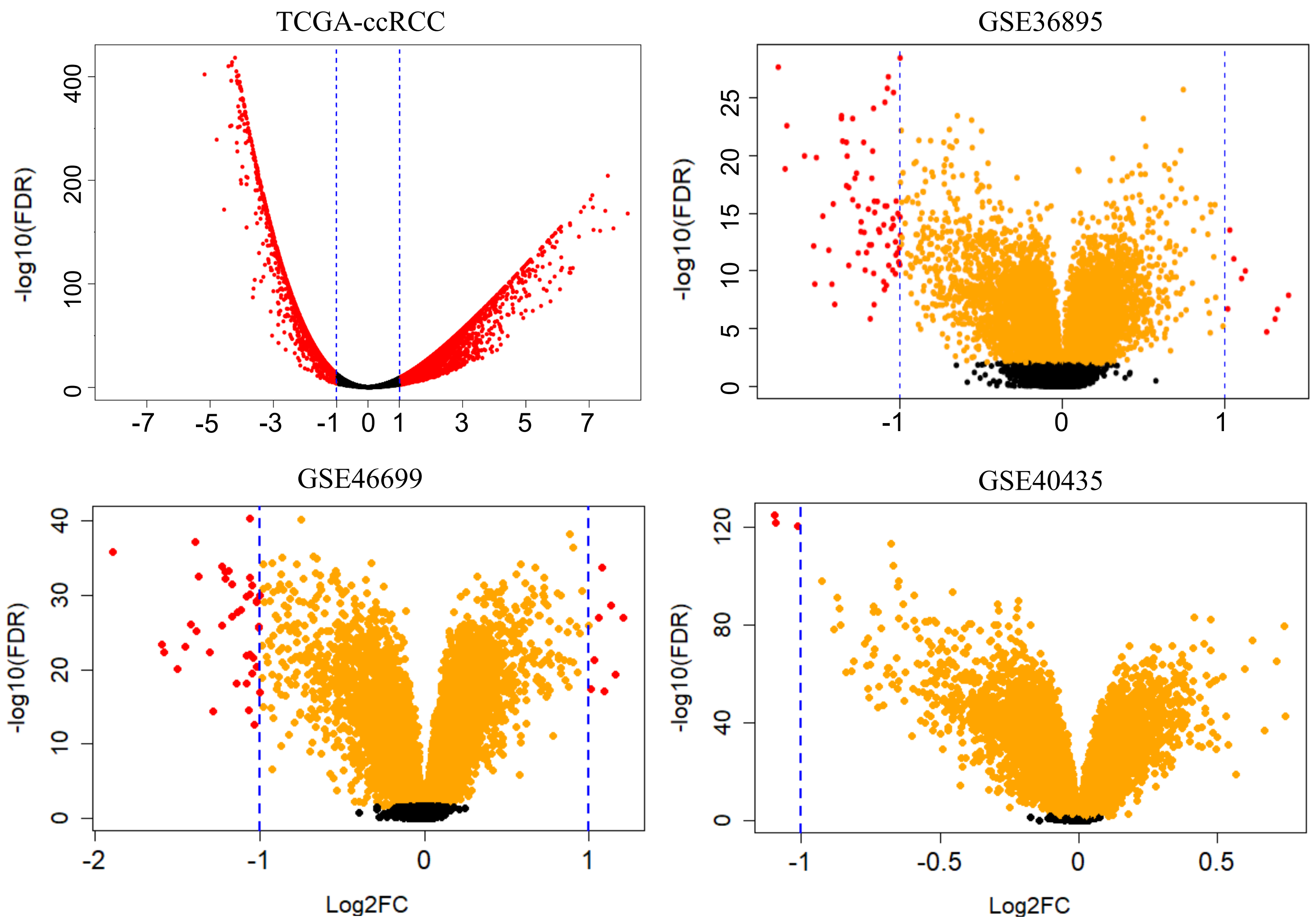

Supp. Figure 1. The volcano plots of the expression of four ccRCC data sets.

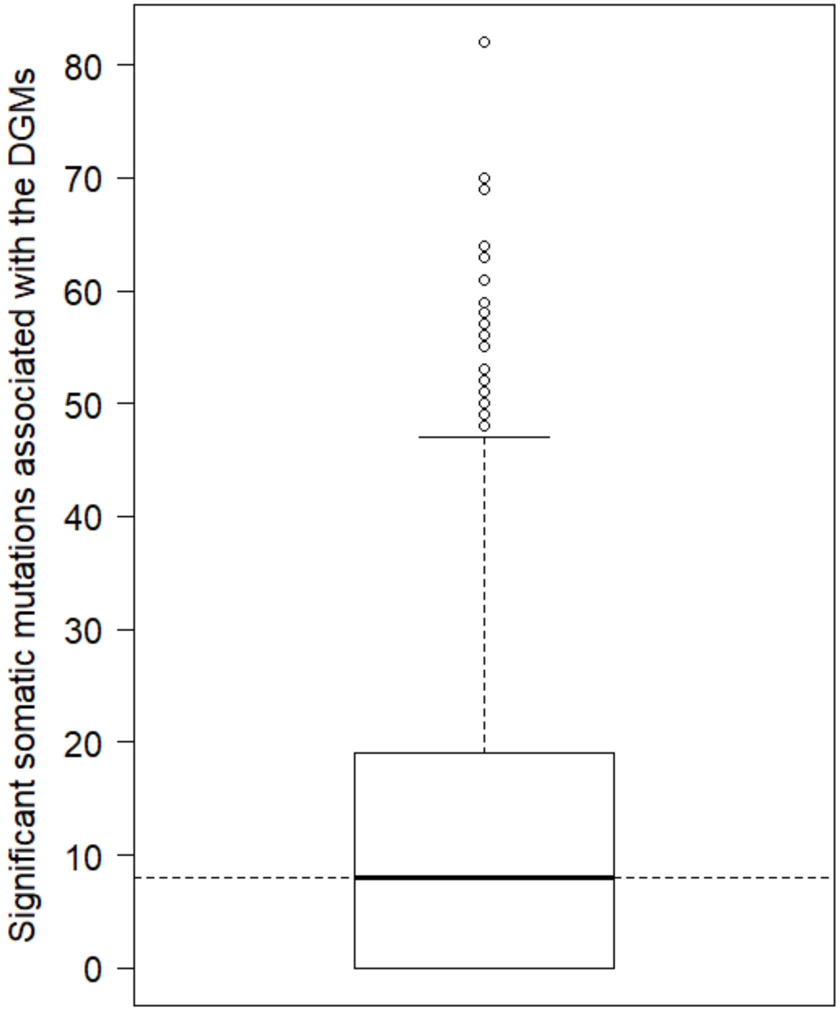

Supp. Figure 2 The box plot of the somatic mutations that were associated with the DGMs

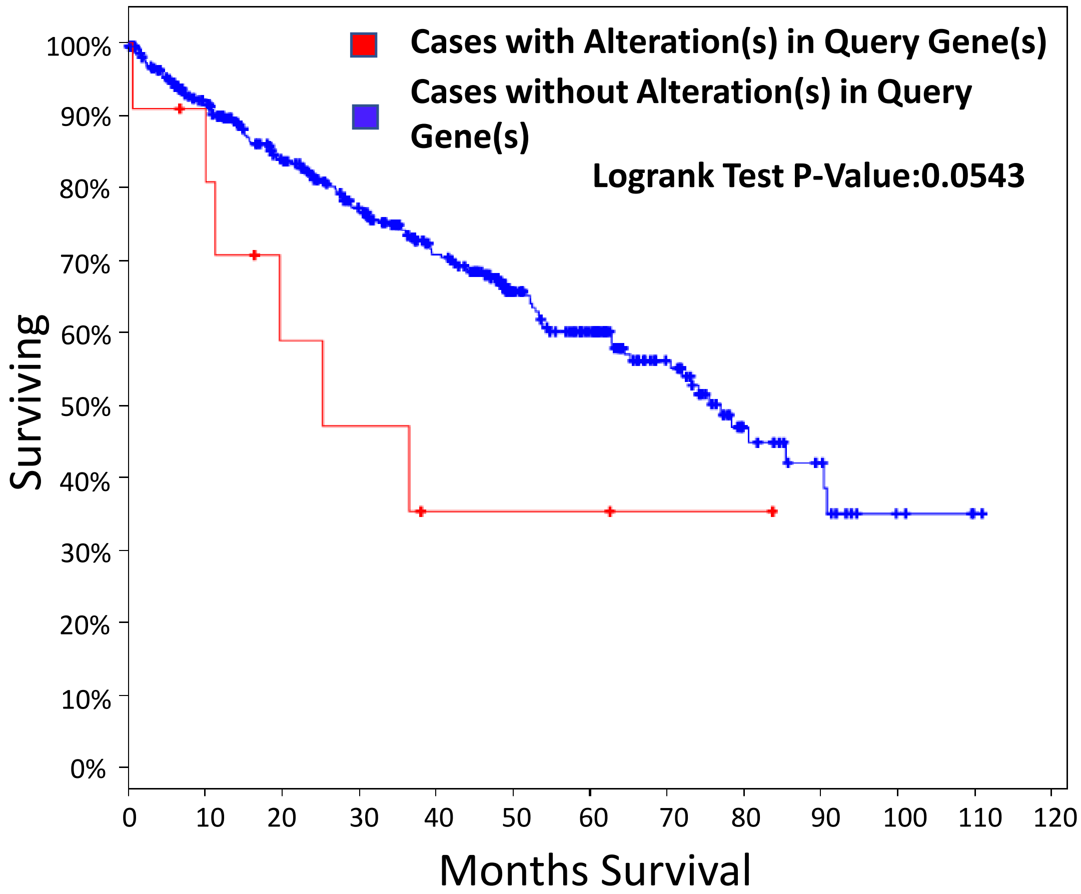

	#total cases	#cases deceased	Median months survival
Cases with Alteration(s) in Query Gene(s)	11	6	25.27
Cases without Alteration(s) in Query Gene(s)	410	136	76.98

Supp. Figure 3 The survival analysis of patients with genetic mutations of NOD2, RRM1, CSRNP1, SLC4A2, TTLL1 and CNTN1
