# Supplementary material for: A Gene Module-Based eQTL Analysis Prioritizing Disease Genes and Pathways in Kidney Cancer
Source: Comput Struct Biotechnol J. 2017 Oct 10;15:463–70. doi: 10.1016/j.csbj.2017.09.003 (PMC5683705; doi:10.1016/j.csbj.2017.09.003)
Supplement: Supplementary file 2 — Supplementary Tables [file mmc2.docx]

**Supp. Table 1 The top significant biological process term of DGMs (P < 0.05)**

| **Biological Process Terms** | **DGMs enriched of the biological process** | |
| --- | --- | --- |
|  | **Number** | **Percentage** |
| response to water | 323 | 30.3% |
| chemosensory behavior | 320 | 30.0% |
| nerve growth factor signaling pathway | 302 | 28.4% |
| programmed cell death involved in cell development | 300 | 28.2% |
| detection of temperature stimulus involved in sensory perception | 298 | 28.0% |
| detection of temperature stimulus involved in sensory perception of pain | 298 | 28.0% |
| Sertoli cell development | 296 | 27.8% |
| behavioral response to pain | 295 | 27.7% |
| cellular response to nicotine | 295 | 27.7% |
| axonogenesis involved in innervation | 294 | 27.6% |
| behavioral response to chemical pain | 294 | 27.6% |
| behavioral response to formalin induced pain | 294 | 27.6% |
| detection of mechanical stimulus involved in sensory perception of pain | 294 | 27.6% |
| olfactory nerve development | 294 | 27.6% |
| response to hydrostatic pressure | 294 | 27.6% |
| detection of temperature stimulus | 280 | 26.3% |
| Sertoli cell differentiation | 278 | 26.1% |
| positive regulation of synaptic transmission, glutamatergic | 275 | 25.8% |
| sympathetic nervous system development | 224 | 21.0% |
| neurotrophin TRK receptor signaling pathway | 219 | 20.6% |
| sensory perception of temperature stimulus | 209 | 19.6% |
| detection of mechanical stimulus involved in sensory perception | 201 | 18.9% |

**Supp. Table 2** The top significant pathways of DGMs (P < 0.05)

| **Pathway** | **DGMs enriched of the pathway** | |
| --- | --- | --- |
|  | **Number** | **Percentage** |
| Thyroid cancer | 297 | 27.9% |
| Aldosterone-regulated sodium reabsorption | 214 | 20.1% |
| Fatty acid degradation | 196 | 18.4% |
| Valine, leucine and isoleucine degradation | 196 | 18.4% |
| Proximal tubule bicarbonate reclamation | 187 | 17.6% |
| Carbohydrate digestion and absorption | 158 | 14.8% |
| Propanoate metabolism | 154 | 14.5% |
| Bile secretion | 138 | 13.0% |
| Bladder cancer | 136 | 12.8% |
| Vasopressin-regulated water reabsorption | 124 | 11.6% |
| Non-small cell lung cancer | 121 | 11.4% |
| Endometrial cancer | 119 | 11.2% |
| beta-Alanine metabolism | 118 | 11.1% |
| Lysine degradation | 117 | 11.0% |
| Tryptophan metabolism | 116 | 10.9% |
| Salivary secretion | 114 | 10.7% |
| Gastric acid secretion | 112 | 10.5% |
| Protein digestion and absorption | 107 | 10.0% |
| Butanoate metabolism | 103 | 9.7% |
| Cardiac muscle contraction | 100 | 9.4% |
| Pathways in cancer | 99 | 9.3% |
| Endocytosis | 95 | 8.9% |
| Melanoma | 94 | 8.8% |
| Pancreatic cancer | 91 | 8.5% |
| MAPK signaling pathway | 90 | 8.5% |
